# Epigenomics and immunotherapeutic advances in pediatric brain tumors

**DOI:** 10.1038/s41698-021-00173-4

**Published:** 2021-04-30

**Authors:** Malak Abedalthagafi, Nahla Mobark, May Al-Rashed, Musa AlHarbi

**Affiliations:** 1 Genomics Research Department, Saudi Human Genome Project, King Fahad Medical City and King Abdulaziz City for Science and Technology, Riyadh, Kingdom of Saudi Arabia; 2 Department of Paediatric Oncology Comprehensive Cancer Centre, King Fahad Medical City, Riyadh, Kingdom of Saudi Arabia; 3 Department of Clinical Laboratory Sciences, College of Applied Medical Sciences, King Saud University, Riyadh, Kingdom of Saudi Arabia; 4 Chair of Medical and Molecular Genetics Research, Department of Clinical Laboratory Sciences, College of Applied Medical Sciences, King Saud University, Riyadh, Kingdom of Saudi Arabia

**Keywords:** CNS cancer, Molecular medicine, Cancer genomics

## Abstract

Brain tumors are the leading cause of childhood cancer-related deaths. Similar to adult brain tumors, pediatric brain tumors are classified based on histopathological evaluations. However, pediatric brain tumors are often histologically inconsistent with adult brain tumors. Recent research findings from molecular genetic analyses have revealed molecular and genetic changes in pediatric tumors that are necessary for appropriate classification to avoid misdiagnosis, the development of treatment modalities, and the clinical management of tumors. As many of the molecular-based therapies developed from clinical trials on adults are not always effective against pediatric brain tumors, recent advances have improved our understanding of the molecular profiles of pediatric brain tumors and have led to novel epigenetic and immunotherapeutic treatment approaches currently being evaluated in clinical trials. In this review, we focus on primary malignant brain tumors in children and genetic, epigenetic, and molecular characteristics that differentiate them from brain tumors in adults. The comparison of pediatric and adult brain tumors highlights the need for treatments designed specifically for pediatric brain tumors. We also discuss the advancements in novel molecularly targeted drugs and how they are being integrated with standard therapy to improve the classification and outcomes of pediatric brain tumors in the future.

## Classification of malignant pediatric brain tumors

Pediatric and adult brain tumors typically emerge from different tissues, and treatments that are relatively well tolerated by the adult brain (such as radiation therapy) may interfere with brain development in children, especially those younger than the age of five^1^. Furthermore, since most treatments were developed for adults, adult and pediatric tumors respond differently to standard therapies. Thus, current treatment options for pediatric brain tumors have limited efficacy due to the different responses to available therapies.

Traditionally, central nervous system (CNS) tumors are classified according to their histological characteristics, but advances in genomic sequencing technologies have allowed molecular profiling that has changed the classification of brain tumors^1^. Molecular profiling and high-throughput methods such as next-generation sequencing (NGS) have resulted in the development of highly targeted molecular therapies. These methods provide insights into disease mechanisms and genomic biomarkers for a precise diagnosis, which allows physicians to design individualized treatments and clarify the differences between pediatric and adult brain tumors.

Molecular profiling has revealed significant differences between adult and pediatric brain tumors, despite having similar histology. For example, groundbreaking research revealed mutations in histone H3 in pediatric high-grade glioma (pHGG); the majority of histone H3 mutations were K27M mutations in which lysine 27 is substituted by methionine^2^. These were the first histone H3 mutations reported in cancer^2,3^. Furthermore, a majority of gliomas with histone mutations cluster with mutations in ATRX (α-thalassemia/mental retardation syndrome X-linked), DAXX (death-domain associated protein), and p53^3^. H3 histone mutations occur in over 50% of pHGGs but in less than 0.2% of adult HGGs^4^.

In 2016, the World Health Organization (WHO) consensus definitions of brain tumors were modified to include a combination of phenotypic and genotypic parameters^1^. The relatively newly described phenotypic heterogeneity now explains the differences in therapeutic responses of tumors with similar histology. The molecular characterization of brain tumor subtypes is ongoing but has already led to the development and use of targeted therapeutics that supplement or replace older cytotoxic approaches.

## Common types of malignant brain tumors

**Fig. 1:**
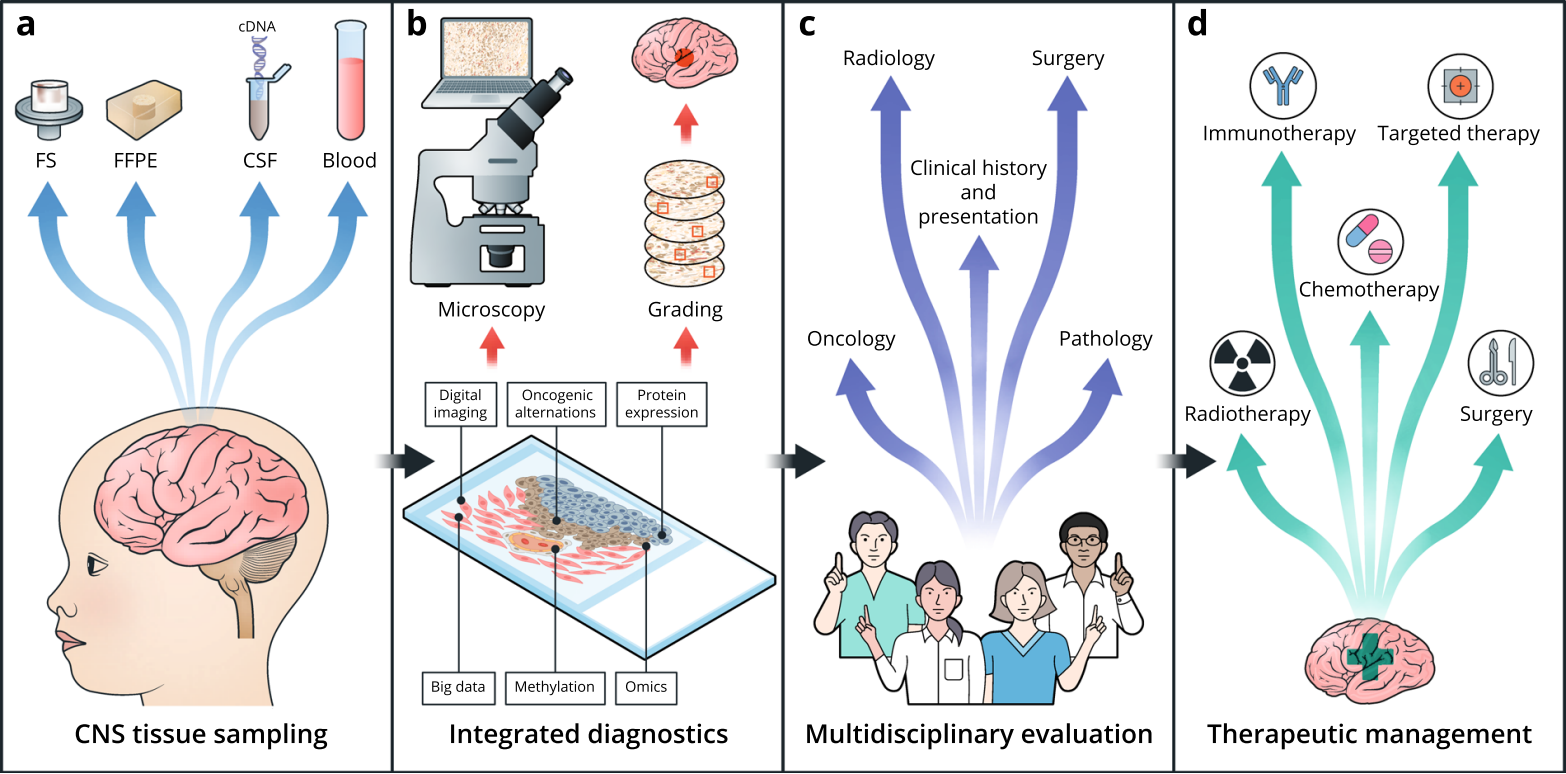
Multistep process for an integrated diagnostic and therapeutic workflow in pediatric neuro-oncology. **a** Obtaining diagnostic samples. **b** Appliying modern diagnostic platforms. **c** Integrated diagnostic in treatment decsions. **d** Using modern theraputic.

The historical classification of CNS tumors was based on histology, anatomical location, and morphologic similarity of tumors to specific cell types in the healthy or developing brain. Molecular profiling along with standard histology allows classifications to align treatment responses and prognoses, as described in the 2016 WHO Classification of Tumors of the Central Nervous System. Figure 1 details the process involved in diagnosing and treating pediatric brain tumors, which includes both histopathological and molecular-based analyses. However, as this classification is not ideal for pediatric tumors, a new WHO classification dedicated to pediatric tumors is in progress^1^.

In adults, there are over 120 histologically different brain tumors grouped into two general types: primary and metastatic. Primary brain tumors originate directly from brain tissues and are subcategorized as glial or nonglial and benign or malignant. Glial tumors are composed of glial cells, while nonglial tumors develop on or in brain structures, including nerves, blood vessels, and glands. Gliomas are the most common type of brain tumor in adults. Based on their histology, glial cells are subdivided into astrocytes, ependymal cells, and oligodendroglial cells. Unlike primary tumors, metastatic or secondary brain tumors arise elsewhere in the body, such as the breast or lungs, and migrate to the brain.

Tumors are also characterized by “grade” (from I to IV); the grade is a “malignancy scale” based in part on histological grading. Histological grading can be used to predict the biological activity of a neoplasm and thereby influences treatment decisions^1^. Low-grade (grades I and II) tumor cells tend to grow and spread slowly, and high-grade (grades III and IV) tumor cells are malignant, fast-growing, and characterized by an abnormal histology, typically with anaplastic (undifferentiated) cells.

Pediatric brain tumors generally fall into different categories than adult brain tumors. The most common pediatric tumors are *high-grade gliomas, medulloblastomas, low-grade gliomas (astrocytic, oligodendroglial, and mixed glial-neuronal), ependymomas, and brainstem gliomas/diffuse intrinsic pontine gliomas (DIPGs)*.

*Medulloblastoma* is a high-grade tumor that usually arises in the cerebellum and is the most common malignant pediatric CNS tumor. Medulloblastoma was originally classified as a glioma, but it is now categorized as a primitive neuroectodermal tumor (PNET). Medulloblastoma accounts for ~20% of all childhood brain cancers and ~63% of intracranial embryonal tumors^5–7^. Medulloblastomas are more common in boys than girls and usually occur between the ages of 2 and 6^1^.

Medulloblastoma is divided into four molecular subgroups: Wingless [WNT]; Sonic Hedgehog [SHH]; group 3 [G3]; and group 4 [G4]. Each subgroup is further divided into several subsets^8,9^. The WNT subgroup has mutations in WNT-α (70%) and WNT-β (30%)^6^. WNT-α mutations occur mostly in children; these patients face an excellent prognosis, as the overwhelming majority of patients with WNT medulloblastoma are disease-free 5 years post diagnosis^10^. Similarly, the 5-year survival rate for SHH medulloblastoma is 80%, and the 5-year survival rate for group 4 medulloblastoma is between 75% and 90%. However, patients with group 3 medulloblastoma face a worse prognosis and a 5-year survival rate of 20–30%^9^, which highlights the necessity for molecular profiling in the diagnosis of brain tumors. Two medulloblastoma tumors may not be molecularly similar, necessitating opposing treatment approaches. Recently, DNA methylation and gene expression data, combined with the integration of somatic copy number alterations and clustered clinical features, revealed 12 different subtypes of medulloblastoma^11^. Over 30% of medulloblastoma samples contained mutations, deletions, or amplifications of genes encoding epigenetic regulators across all four subgroups, and these aberrations can be used to stratify medulloblastomas^12^.

pHGGs are biologically distinct from adult HGGs^13–15^. HGGs refer to malignant, diffuse, infiltrating astrocytic tumors of WHO grade III (anaplastic astrocytoma) and grade IV (glioma). The histological appearance of pHGG is the same as that of adult malignant gliomas. Compared with adult HGGs, pHGGs are more frequently associated with platelet-derived growth factor/platelet-derived growth factor receptor (PDGF/PDGFR) genomic alterations and mutations in the histone H3.3 gene and less frequently with PTEN and EGFR genomic alterations^4,16^.

The classification of pHGGs is based on molecular subgroups and significant clinical correlations (i.e., age, anatomical location, and prognosis). The major molecular groups are as follows: (1) histone mutations and H3.K27‑mutated midline and H3.G34‑mutated hemispheric pHGG; (2) rare isocitrate dehydrogenase (IDH)‑mutated pHGG (mainly in adolescents); and (3) wild-type H3‑/IDH pHGG, a heterogeneous group that remains to be fully characterized^16^. Overall, histone mutations represent slightly more than half of all childhood pHGG cases.

The largest pHGG dataset to date was used in a retrospective study that revealed ten subgroups based on specific genes and processes^4^ and showed that histone mutations cosegregate with distinct modifications and downstream pathways. This study demonstrated specific changes in genes encoding histone H3 mutations and reported that histone wild-type tumors and tumors with BRAF mutations are less aggressive than tumors with different mutational profiles.

Most pediatric gliomas are grade 1 or 2 low-grade gliomas, but some progress rapidly to grade 3 or 4 as pHGG^17^. Astrocytomas are the most common glioma and represent ~25% of all primary brain and spinal cord tumors in children^18,19^. While astrocytomas are more prevalent in adults as HGGs, in children, most of these tumors are low-grade^20^. Grade I pilocytic astrocytoma is more common in children than in adults and has an excellent prognosis^1,20^. Pilocytic astrocytoma is a “single-pathway” disease, with mutations primarily in the mitogen-activated protein kinase (MAPK) pathway^20,21^.

*Oligodendrogliomas* are derived from myelin-producing cells and have unique genetic characteristics and a better response to chemotherapy than other gliomas. These tumors are classified by a grade of II or III and are histologically similar to oligoastrocytomas. Pediatric oligodendrogliomas differ significantly from adult oligodendrogliomas based on factors such as race, tumor size, tumor location, and tumor grade. These key prognostic factors play different roles in pediatric and adult tumors^22^. In children, oligodendrogliomas are often wild-type IDH, with mutations in the TERT (human telomerase reverse transcriptase) promoter and 1p/19q codeletion, in contrast to those in adults^23^. They are classified as oligodendrogliomas based primarily on histology^24^. Pediatric tumors usually have single pathogenic alterations in the fibroblast growth factor receptor 1 (FGFR1) oncogene^25^.

*Ependymomas* are rare primary gliomas of the CNS that originate from ependymal cells lining the ventricular system. Ependymomas arise within three main areas: the posterior fossa (PF) and supratentorial (ST) regions of the brain and the spine. Based on histopathology, the WHO classifies ependymomas into four subtypes: subependymoma and myxopapillary ependymoma (grade I); ependymoma (grade II); ependymoma, *RELA*-fusion-positive (grade II or III); and anaplastic ependymoma (grade III). Ependymomas can be grade I (more common in adults) or II or III (malignant)^1^. The tumor location varies by age, as pediatric ependymomas originate in the brain, and adult ependymomas originate in the spine^26^. Survival is poorer in children than in adults^27^. Tumors in each area have discrete genetic, epigenetic, and cytogenic abnormalities and multiple molecularly distinct subsets within each region^26,28^. Pediatric posterior fossa ependymomas (pPFEs) are mostly found in young children and have a considerable risk of local recurrence even after resection and postoperative radiotherapy. An important part of the treatment modality includes repeated resections, which are important for tumor control. While the majority of pPFE patients succumb to the disease, some survive with excellent functional outcomes^29^. Posterior fossa ependymomas have global DNA hypomethylation and CpG island hypermethylation, resulting in the silencing of many genes that play a significant role in chromatin modification.

*DIPG* is a high-grade brainstem glioma^30^ accounting for 75–80% of all pediatric brainstem tumors^31^. pHGG tumors with mutant H3, in which lysine is substituted by methionine at position 27 in histone 3.1, 3.2, or 3.3 (H3 K27M), are most exclusively found in midline structures. Eighty percent of DIPG patients have this mutation, which resulted in the WHO classification of these tumors as a new group of diffuse midline glioma (DMG) (i.e., H3 K27M‐mutant). The point mutation in the histone H3 K27M gene is a defining feature of DIPG^32^, and this mutation specifically impacts the epigenome^33^. These tumors have an extremely poor prognosis, as their intrinsic nature makes it impossible to perform resection. They are also unresponsive to chemotherapy, and radiotherapy is minimally effective^30,34^. DIPG also has genomic alterations in tumor protein p53 (*TP53*), platelet-derived growth factor receptor alpha (*PDGFRA*), and activin A receptor type I (*ACVR1*), among other genes^35^. The diverse array of mutations contributes to significant intertumoral heterogeneity in DIPG, complicating treatment strategies^36^.

Knockdown of the histone H3 K27M mutation in animal models using DIPG xenografts restored the K27M-dependent loss of H3 K27M and delayed tumor growth^36^. Knockdown experiments illuminated the effects of the K27M mutation on the transcriptome and epigenome and pointed to genes associated with nervous system development.

## Epigenetic modifications

Gene expression profiles associated with cancer reflect germline and somatic mutations. In contrast, epigenetic mechanisms regulate gene expression without altering the original DNA sequence. Epigenetic modifications are reversible structural alterations of the nucleic acid and histone proteins that constitute the nucleosome. The reversibility of epigenetic modifications makes them an attractive drug target. The identification of genes that control epigenetic changes has produced novel targets for cancer treatments, particularly treatments for CNS tumors^37,38^.

DNA in chromatin is wrapped around histone proteins organized in nucleosomes. A nucleosome has 147 DNA base pairs around an octamer of four core histone proteins. Each histone octamer contains two copies of each of the histone proteins H2A, H2B, H3, and H4. N-terminal histone tails protrude from nucleosomes into the nuclear lumen and are accessible for modifications. Histone H1 associates with the linker DNA between repeating nucleosomes. Histone modifications regulate chromatin structure and gene accessibility to both the DNA and histone tails. Primary epigenetic mechanisms include discrete but interrelated processes, including DNA methylation, histone density and posttranslational modifications, and RNA-based mechanisms (e.g., microRNAs). When tumor suppressors are the target of DNA methylation or histone deacetylation, they are silenced; tumor suppressor silencing contributes to cancer development^39,40^.

Epigenetic regulation involves a sequence of enzymes. “Writers” add groups (e.g., methyl, acetyl, and glycans), “erasers” remove posttranslational modifications, and “readers” recognize epigenetic markers and regulate epigenetic effects. The protein complexes that mediate the movement of nucleosomes along chromatin are known as “movers.” Currently, DNA methyltransferases (DNMTs) and histone deacetylases (HDACs) are the most prominent targets of therapeutics^41,42^, and the best characterized “epidrugs” are DNMT inhibitors (DNMTis) and HDAC inhibitors (HDACis)^43^.

Epigenetic mechanisms also include microRNA activity. MicroRNAs are small noncoding RNA molecules that are involved in RNA silencing and the posttranscriptional regulation of gene expression^44^. MicroRNAs are differentially expressed in many brain tumor subtypes and can be used as biomarkers for CNS tumor diagnoses^45^. For example, the H3F3A mutation is associated with changes in microRNA levels^46^. MicroRNAs have been evaluated as mediators of therapeutic resistance in childhood CNS tumors^47^. However, in contrast to epigenetic modifiers, therapeutic options to inhibit microRNAs have not been developed, and no existing pediatric oncology study has investigated microRNA-based treatments^48,49^.

## Epigenetics and brain cancer

The association between epigenetic changes and cancer development has become key to understanding cancer development and to developing novel cancer treatments. Epigenetic changes are essential to malignant transformation, as^48,49^ tumor tissues exhibit abnormal patterns of methylation compared to healthy tissues^41,49,50^. Epigenetic profiles may be useful for: (a) potential drug targets; (b) cancer prognostics; (c) tumor classification; and (d) the basic science of tumor development^49^.

Epidrug treatments aim to reverse epigenetic changes such as DNA methylation to induce tumor suppressor re-expression or reverse an immune-suppressed environment^50^. Profiling each tumor for epigenetic changes (in addition to gene modifications) will improve stratification in clinical trials and predictions of responses to therapy^51^. Tumor cells also use epigenetic mechanisms to escape chemotherapy and host immune surveillance, so improving our understanding of these mechanisms will improve treatment outcomes^50^.

DNA methylation is the most studied epigenetic modification^52^. Both DNA and histones can be methylated, and there is also evidence for crosstalk between histone modifications and DNA methylation^39,53^. In mammals, DNA methylation occurs primarily by the covalent modification of cytosine residues in CpG dinucleotides (CpG islands). When a DNA residue is methylated, the enzyme DNMT utilizes the cofactor s-adenosylmethionine (SAM) as a methyl donor. There are three main types of DNMTs: DNMT1, DNMT2, and DNMT3 (DNMT3a and DNMT3b). DNA methylation controls gene expression by contributing to changes in chromatin structure, DNA conformation, DNA stability, and interactions between DNA and proteins. DNA methylation characteristically represses gene transcription and can silence tumor suppressors when a gene promoter is methylated^54^.

DNMT inhibition is an effective method of preventing abnormal DNA hypermethylation. However, DNMTs are ubiquitous, and targeting methyltransferase enzymes has limitations, including a lack of tumor specificity and a tendency to cause global hypomethylation of the genome. Despite these drawbacks, some DNMT inhibitors, such as 5-azacytidine and decitabine (5-aza-2′-deoxycytidine), have been approved by the FDA^41,55,56^. These are often used in combination with standard therapies, allowing lower doses and less-severe side effects for individual drug regimens.

Histone methylation most often occurs on the lysine residues of histone tails H3 and H4. H3K4, H3K48, and H3K79 methylation sites are commonly associated with gene activation, and H3K9 and H3K27 methylation sites are commonly associated with gene inactivation^57^.

Histones function to positively and negatively regulate gene expression primarily by posttranslational modifications. Histone acetyltransferases (HATs) and HDACs are responsible for maintaining histone acetylation levels. Among the histone-modifying enzymes, HATs and HDACs are the most studied targets for chromatin remodeling, gene expression, and chemotherapies.

HDACs are critical to our understanding of oncogenesis because they can silence tumor suppressor genes and genes involved in apoptosis^58^ and remove acetyl groups from histones, which allows histones to wrap the DNA more tightly and thus prevent transcription. HDACs are often upregulated in cancers^59,60^, which makes them potential therapeutic targets.

### Epigenetic changes in pediatric brain tumors

The use of DNA methylation signatures as part of a combined histology and molecular tumor classification for pediatric brain tumors was first demonstrated by Capper et al.^61^ on the basis that each brain tumor subtype has discrete genetic and epigenetic profiles^34^. The central role of DNA methylation‐based classification is to exclude less-malignant neoplasms with misleading histological features. While adult brain tumors have specific mutational profiles that aid in diagnosis and prognosis, a large proportion of pediatric tumors lack genetic lesions or specific drivers detected by NGS^62–64^. Thus, for pediatric CNS tumors, epigenetic modifications can also serve as therapeutic drug targets. For example, the identification of *IDH1/2* mutations in adult gliomas was a landmark discovery in adult brain tumor management. *IDH1/2* mutations are prevalent (over 80%) and often co-occur with p53 gene (TP53) mutations and total 1p/19q deletions^65,66^. IDH drives methylation by metabolizing isocitrate to α-ketoglutarate (α-KG). Mutations in *IDH1* or *IDH2* reduce α-KG levels due to increased D-2-hydroxyglutarate (2-HG) levels. α-KG is an essential cofactor for specific histone and DNA demethylases, while 2-HG is a competitive inhibitor. Hypermethylated histones and DNA are thought to be a result of *IDH1/2* mutations^67^. In contrast, *IDH1/2* mutations are rare in pediatric glioma; *IDH1* or *IDH2* mutations in children occur in a small proportion (6.25%) of tumors^16,34,68^. Epigenetic profiles of pediatric brain tumors can be more informative than their mutational profiles. Tumors originating in infants, young children, and adolescents have differing epigenetic profiles^64^. For example, the epigenetic profiling of pediatric ependymomas provided guidance for additional molecular tests, especially with respect to the close association of RELA and YAP1 fusion proteins with certain molecular classes, indicating which tumors to test for these gene fusions and in which tumors testing can be omitted^69^. Based on the methylation profiles, the ependymal classes include the known PFA and PFB groups, the previously described supratentorial RELA‐fusion‐positive ependymoma, and three new molecular classes consisting of supratentorial YAP1‐fusion‐positive ependymomas, a benign spinal ependymoma, and a class closely related to the histological group of myxopapillary ependymoma^28^. Additional classifications of ependymomas based on DNA methylation have demonstrated high rates of misdiagnosis when using histopathology alone. Therefore, in the case of ependymomas, the pivotal role of DNA methylation profiling is to validate the histological diagnosis^70^.

Additionally, G-CIMP (glioma-CpG island methylator phenotype) was identified as a distinct subset of human gliomas based on molecular and clinical characteristics^71^; clinically relevant subgroups of adult G-CIMP tumors (G-CIMP-high and G-CIMP-low) have further refined glioma classification independent of grade and histology^72^. However, Jha et al.^73^ found that pediatric glioblastoma has a methylome that is distinct from adult glioblastoma, suggesting that the G-CIMP indicator of glioma prognosis in adult glioblastoma multiforme (GBM) cannot be generalized to pediatric GBM.

There is significant heterogeneity within and between tumor subtypes, and thus, epigenetic profiling and targeted epidrugs could provide effective personalized therapy. Epidrugs act on the enzymes needed for epigenetic modifications, with the current strategy focused on the inhibition of DNMTs and HDACs^74^.

### DNMT inhibitors and pediatric brain cancer

The DNMT inhibitors azacytidine and decitabine are highly effective epigenetic drugs and are commonly used despite their toxicity and poor chemical stability^37,75–77^. They are successfully used in combination with other pharmaceutical agents, such as immune checkpoint inhibitors or chemotherapeutics^37,78^. There are hundreds of clinical trials on DNMTis in many cancers, including the clinical study of pediatric brain tumors treated with the DNMTi 5-azacytidine (NCT03206021), which is a phase 1 trial that combines 5-azacytidine and carboplatin for recurrent/refractory pediatric brain and solid tumors.

### HDAC inhibitors and pediatric brain tumors

HDACis can restore the balance between acetylation and deacetylation and, in addition to changes in chromatin structure, have far-reaching effects on multiple processes^79^. While HDACis have proven effective against brain tumors, the mechanisms of HDACi treatment are complex and not yet completely understood. Due to the functional redundancy of different HDACis, their clinical effectiveness is limited^80,81^. HDAC inhibitors alter gene expression and both histone and nonhistone proteins^82^. Currently, four HDACis have been approved by the FDA, vorinostat, romidepsin, belinostat, and panobinostat, and others are in the clinical trial stage in combination with standard therapy^83^.

Vorinostat (suberoylanilide hydroxamic acid (SAHA)) acts on class I and class II HDACs and is the most commonly used HDACi. Since it showed poor efficacy in solid tumors (i.e., mesothelioma) as a single agent, most clinical studies employ combination strategies^84^.

Romidepsin inhibits mainly class I HDACs and is a prodrug that is activated in cells. Weak activity was observed on solid tumors, leading to the evaluation of combination strategies in clinical trials.

Belinostat has a broad spectrum of action (class I and class II HDACis). Belinostat was approved by the FDA in 2014. The activity of belinostat alone in solid tumors is low, but it is being assessed in combination with chemotherapeutic agents (e.g., cisplatin and carboplatin)^84^.

Panobinostat is a pan-HDACi. It was approved by the FDA in 2015 for multiple myeloma and has since been evaluated in many phase II or III clinical trials on different cancers to evaluate its efficacy alone or in combination.

Brain tumors seem to be vulnerable to treatment with HDACis, as has been shown for glioblastoma (15, 16), atypical teratoid/rhabdoid tumors, and medulloblastoma (17–19). HDACi sensitivity appears to be due to mutations in the HDACi resistance mechanism^80^. HDACis were used in several completed trials and are being used in ongoing clinical trials on pediatric brain tumors (Table 1); most employ combination therapies such as chemotherapy (NCT00994500, NCT00867178, and NCT01076530; all completed), a proteasome inhibitor (marizomib; NCT04341311; ongoing), and radiation (NCT00867178; completed). Interestingly, the combination of panobinostat and marizomib has been shown to cease tumor-cell ATP production by DIPG cell mitochondria^85^.

**Table 1:** Selected clinical trials on HDACis in pediatric neuro-oncology.

Therapeutic HDACi	Combination agents	Age (years)	Condition (tumor type)	Phase	Clinical trial identifier
Entinostat	None	1–21	Recurrent primary CNS neoplasm	I	NCT02780804
Panobinostat	Marizomib	up to 21	DIPG, glioma	I	NCT04341311
Vorinostat	Radiation + isotretinoin + chemotherapy	2–47 months	CNS embryonal tumors	I	NCT00867178
Romidepsin	None	up to 21	CNS tumors + others	I	NCT00053963
Vorinostat	Temozolomide	1–21	Primary CNS tumors	I	NCT01076530
Vorinostat	Bortezomib	1–21	Primary CNS tumors	I	NCT00994500

### Limitations of epigenetic therapies

Responses to HDACis vary among different brain tumor types^80^. Human cells possess natural resistance to HDACis, decreasing their toxic effects, and may eventually develop resistance to HDACi therapies^50,86^. However, this natural resistance to HDACis may be disrupted by the mutations inherent in tumors, which makes such tumors sensitive to HDACis^80^.

HDACs are ubiquitous and are not limited to tumors^87^. HDACis to specific HDAC enzymes have proven difficult to develop due to the overlapping functions of HDACs. Most HDACis affect either all or at least a range of HDACs (21). Thus, the inhibition of several HDACs may occur and cause side effects; therefore, the targeted blockade of a single HDAC might be more desirable.

## Immunotherapy

Immunotherapy aims to treat cancer by generating or enhancing an immune response against the tumor. Immunotherapy can be broadly categorized as monoclonal antibody (mAb) therapy and adoptive cellular therapy (ACT). Advances in immunotherapy include immune checkpoint blockade, chimeric antigen receptor (CAR)-T cell therapy, vaccine therapy^88,89^, and vaccine and oncolytic virus therapy^90–92^.

While many of the principles of brain tumor biology and immunology are the same for pediatric and adult tumors, there are significant differences between the two. Compared to adult brain tumors, pediatric brain tumors have a lower mutational load and corresponding neoantigens and a tumor microenvironment that is less immunosuppressed. Therefore, immunotherapies developed for adult brain cancer are often not as effective for pediatric brain cancer. There are relatively fewer trials on immunotherapy for pediatric brain tumors than for adult brain tumors, but there is work being done towards improving immunotherapy for pediatric brain tumors^88–90^.

### General considerations and factors that influence immunotherapy for pediatric brain tumors

There are similarities and differences that determine the effectiveness of immunotherapy in adult and pediatric brain cancers.

A specialized interface between the blood circulation and CNS surrounds the brain. This interface was thought to be a barrier and to confer “immune privilege,” although more recent evidence suggests that it is a “gatekeeper” and not a complete barrier^93–95^. Some substances can cross the BBB, as the BBB becomes leaky in the presence of a tumor, and radiation may alter BBB permeability^93,96^. Low-intensity pulsed ultrasound (LIPU) seems to “open” the BBB in preclinical models and humans, and can enhance drug delivery to the brain^96^. Although promising, methods to control the BBB still require evaluation and standardization, particularly because there is very little information on the pediatric BBB and its contribution to immunotherapy resistance.

Responses to specific therapies differ between adults and children in many cancers, including brain cancer^97^. This is most likely because children have fewer genetic mutations and more epigenetic alterations than adults^3,9,98,99^. While nonsynonymous mutations that generate neoantigens are optimal immunotherapy targets, neoantigens are less prevalent in tumors with low mutational burdens, such as pediatric brain tumors. Tumor mutational burden (mutational load) is a quantitative measure of the total number of mutations per coding area of a tumor genome; it is correlated with the sensitivity to immune checkpoint inhibitors^100^.

Interestingly, the mutational load increases with age; adolescent brain tumors have an intermediate mutational load and immunogenicity between children and adults^101^. The low mutational load (and corresponding neoantigen level) in pediatric brain tumors is a central issue for immunotherapy since appropriate tumor antigens are needed as targets to induce an immune response against tumors.

“Hot” tumors have more tumor-infiltrating lymphocytes (TILs), particularly T cells, than “cold” tumors^5^. In adult glioma, higher numbers of infiltrating CD8+ T cells are associated with longer survival^102^. Pediatric brain tumors are generally immunologically “cold” and lack T cell infiltration^103,104^. Tumor neoantigens, activating cytokines, the tumor vasculature, and integrins all contribute to T cell homing^5^; brain tumors have a unique extracellular matrix that prevents T cell migration^105^. Immunotherapy aimed at promoting or enhancing the T cell response will be ineffective when T cell infiltration is low^106^. Small numbers of immune cell infiltrates, along with a low mutational burden, are believed to significantly reduce the immunogenicity of pediatric brain tumors^106^.

Newer approaches are directed at combining therapies that increase immune system function in the tumor microenvironment. For example, radiation can increase the number of tumor antigens recognized by the immune system and upregulate the expression of the major histocompatibility class I molecule on tumor cells^107^. Thus, radiation is often combined with therapeutic agents (Table 2). Additionally, DNMTis and HDACis enhance the expression of genes responsible for the expression of tumor antigens, antigen processing and presentation machinery, and immune-related genes to facilitate the conversion of “cold” tumors to “hot” tumors^108^.

**Table 2:** Selected clinical trials on immunotherapy in pediatric neuro-oncology.

Immunotherapy category	Investigational agent	Age (years)	Condition (tumor type)	Phase	Clinical trial identifier
CAR T cells	HER2-specific CAR-T cells	1–26	HER2-positive recurrent or refractory CNS tumors	I	NCT03500991
	IL13Rα2-specific CAR-T cells	12–75	Recurrent or refractory high-grade glioma (HGG)	I	NCT02208362
	B7H3-specific CAR-T cells (SCRI-CARB7H3(s))	1–26	Diffuse intrinsic pontine glioma (DIPG), diffuse midline glioma (DMG), and recurrent or refractory CNS tumor	I	NCT04185038
	EGFR806-specific CAR-T cells	1–26	Recurrent or refractory pediatric CNS tumors	I	NCT03638167
	HER2-specific CAR-T cells	3 +	Recurrent or refractory brain tumor	I	NCT02442297
Checkpoint blockade	IDO pathway inhibitor + indoximod and temozolomide	3–21	Progressive malignant brain tumors (including DIPG)	I	NCT02502708
	Pembrolizumab	1–29	Recurrent, refractory HGG, DIPG, hypermutated tumors, ependymoma, medulloblastoma	I	NCT02359565
	Pidilizumab (MDV9300) + radiation or cyclophosphamide	3–21	DIPG	I/II	NCT01952769
	Nivolumab	1–18	Hypermutant solid malignancies with biallelic mismatch repair deficiency (bMMRD)	I/II	NCT02992964
	Nivolumab and nivolumab + ipilimumab	0.5–21	DIPG, HGG, medulloblastoma, ependymoma, other high-grade CNS tumors	1b/II	NCT03130959
Vaccine/viral	ADV-tk + prodrug therapy + radiation (viral gene therapy)	3–21	Malignant glioma (grade III or IV); recurrent ependymoma	I	NCT00634231
	Ad-RTS-hIL-12 + veledimex (viral gene therapy)	Up to 21	DIPG and malignant brain tumors	I/II	NCT03330197
	PEP-CMV peptide vaccine (human pp65-CMV glycoprotein B + KLH)	3–35	Recurrent medulloblastoma and malignant glioma	I	NCT03299309
	Modified measles vaccine (MV-NIS)	1–39	Recurrent medulloblastoma or ATRT	I	NCT02962167
	DNX-2401 oncolytic virus targeting an abnormal RB pathway	1–18	DIPG	I	NCT03178032
	H3.3K27M peptide vaccine + tetanus toxoid peptide + poly-ICLC	3–21	HLA-A201+ in newly diagnosed DIPG and H3.3K27M-positive glioma	I/II	NCT02960230
	Glioma-associated antigen/peptide vaccine (EphA2, IL-13Rα2, survivin) + poly-ICLC	1–21	HLA-A2+ children with DIPG or recurrent or HGG	Pilot	NCT01130077
	Tumor lysate-pulsed DC vaccine + imiquimod	13–99	Glioblastoma multiforme, anaplastic astrocytoma, HGG	I	NCT01808820
	Heat shock protein personalized peptide complex-96 (HSPPC-96) vaccine + radiation	3–21	Newly diagnosed or recurrent HGG	I	NCT02722512
	Neo-epitope personalized synthetic long personalized peptide vaccine	0–21	Recurrent malignant CNS tumors	I	NCT03068832
	Cytomegalovirus (CMV)-specific peptide vaccine (PEP-CMV)	3–35	Medulloblastoma, brain tumor, malignant glioma	I	NCT03299309
	CMV-RNA-pulsed DCs + GM-CSF + tetanus toxoid	Up to 35	Glioblastoma, glioma, medulloblastoma, brain tumor	I	NCT03615404
	DC vaccine + temozolomide + radiation	13–70	Glioblastoma	II	NCT02772094
	HSV G207 (oncolytic virus) + radiation	3–18	Supratentorial neoplasms, glioma, glioblastoma, anaplastic astrocytoma, cerebral primitive neuroectodermal tumor, embryonal tumor	I	NCT02457845
	Nimotuzumab (EGFR antibody) + radiochemotherapy	3–12	Brainstem neoplasm	II	NCT02672241
	^131^I-8H9 (8H9 antibody attached to the radiation dose)	Any	Brain and CNS tumors	I	NCT00089245
	^131^I-3F8 (3F8 antibody attached to the radiation dose)	3+	Brain and CNS tumors	II	NCT00058370
	TTRNA-DC vaccine + GM-CSF + TMZ (after chemoradiation)	3–21	Malignant glioma, HGG	I	NCT03334305
	K27M peptide vaccine + nivolumab	3–21	H3.3K27M-positive DIPG, glioma (non-DIPG), DMG	I/II	NCT02960230
	ONC201 (DRD2 antagonist)	2–18	H3.3K27M-positive DIPG	I	NCT03416530
	Oncolytic HSV-1 G207 + radiation	3–21	HGG	II	NCT04482933

Tumor-driven immune evasion is one of the major obstacles to improving the effectiveness of cancer immunotherapies. Epigenetic modifiers, such as DNMTis, in combination with immunotherapy are under investigation to improve efficacy^43,50,109^. The level of immune suppression varies according to the brain tumor type^110–114^. For example, among pediatric tumors, immune cell infiltration in DIPG tumor tissue is similar to that in nontumor tissue, and immunosuppressive factors such as PD-1 and TGFβ1 are not overexpressed. Histone H3.3-K27M-positive DIPG cells do not repolarize macrophages and are not targeted by activated allogeneic T cells, and NK cells are functional. Thus, the DIPG tumor environment is not highly immunosuppressive, although it lacks effector immune cells^114^. Therefore, the genetic, epigenetic, and immune profiles of each tumor need to be assessed to design an appropriate therapy.

Since brain tumors in children are relatively rare, the number of potential patients for clinical trials is small^115,116^, and these trials are rarely randomized. Collaborative, multicenter trials have successfully increased participant numbers. The term “childhood” is defined in the Clinical Trials database (clintrials.gov) as birth to age 17. However, many pediatric clinical trials include patients over 18 years old, which complicates the interpretation of trial outcomes. For trials restricted to patients under the age of 18, participant numbers of seven were recorded for a completed study in a single center (NCT00107185). Multicenter trials generally have 30 to 50 participants and often include multiple pediatric brain cancer types (e.g., NCT02793466, NCT03206021, and NCT02672241), which can make it challenging to interpret the results. Furthermore, pediatric brain tumors are extremely heterogeneous, so results based on a small number of participants are difficult to interpret without molecular profiling to stratify the patients^51,117,118^.

The majority of patients do not benefit from immunotherapy, as tumors often fail to respond or develop resistance, presenting a significant hurdle to improving the efficacy of immunotherapy. Patients can experience *primary resistance*, where the cancer does not respond to immunotherapy; *adaptive immune resistance*, where the cancer cells adapt to the immune attack; and *acquired resistance*, where the cancer cells initially respond to the immunotherapy, but after a certain interval, they relapse and progress^119,120^.

Multiple mechanisms are involved in resistance to immunotherapy^119–121^; these include the therapeutic agent and partly the genetic, epigenetic, and immune profiles of the tumors. Cancer cells can become resistant to engineered monoclonal antibodies by losing the expression of the target antigen. Additionally, the development of neutralizing antibodies against therapeutic antibodies can decrease the response. In general, tumor resistance does not appear to differ between children and adults^121^.

Medulloblastomas and DIPGs that lack the p53 tumor suppressor often do not express surface MHC1, making them resistant to immune rejection^122^. p53 regulates MHC1 localization by controlling the expression of Erap1 and Tap1, proteins needed for MHC1 translocation to the cell surface. Additionally, Garancher et al.^122^ showed that medulloblastoma and DIPG cells lacking MHC1 are not recognized and killed by CD8+ T cells. In animal models and cells, TNF and lymphotoxin ß receptor (LtßR) antigen rescued MHC1 expression and enhanced the responses to immune checkpoint inhibitors independent of p53^122^. The study revealed p53 as a key regulator of immune evasion. The results provide preclinical evidence that TNF could be used to restore MHC1 and enhance the sensitivity of tumors.

Immunotherapies have fewer long-term toxicities than standard chemotherapy and radiation treatments. As the field of immunotherapy against cancer is relatively new, there is limited information on the short- and long-term effects of immunotherapy in children, and knowledge on side effects has been derived from studies on adults. Potential side effects of checkpoint therapies include chills, fever, headache, myalgias, and fatigue. Acute reactions to monoclonal antibodies are relatively common but easily managed with antipyretics, antihistamines, or corticosteroids. The toxicity of anti-PD1 therapies is often immune related (e.g., pneumonitis, colitis, hepatitis, hypophysitis, autoimmune hemolytic anemia, thyroiditis, and dermatitis)^104,107,123–126^. Most of these side effects respond to steroids and have minimal long-term effects^124^. However, since adoptive T cell therapy uses targets expressed on both tumor cells and healthy tissues, toxicity is a serious concern^127,128^. Adoptive T cell therapy can also cause potentially fatal cytokine release syndrome, which can lead to multiple organ failure^129^.

## Immunotherapy for pediatric brain cancer

Immune checkpoints regulate T cell function by modulating the T cell response. Immune checkpoint pathways can be stimulatory (e.g., via the TNF superfamily and B7-CD28 superfamily) or inhibitory (e.g., via PD-1, CTLA-4, and IDO), and their function maintains control and self-tolerance. Checkpoint signals regulate antigen recognition of the T cell receptor (TCR) during an immune response. Cancer growth is facilitated partly by immune suppression induced by the tumor. Tumors hijack and activate suppressive immune checkpoint pathways to reduce immune responses to the tumor^130–132^. Negative checkpoint regulators on T cells led to the immune eradication of solid tumors in mice, showing the potential for treatment using immune checkpoint inhibition^133^. Therapeutics primarily use immunomodulatory monoclonal antibodies to target inhibitory immune checkpoint molecules such as PD-1, CTLA-4, and IDO.

The most prominent inhibitory checkpoint molecules are cytotoxic T lymphocyte-associated molecule-4 (CTLA-4), programmed cell death receptor-1 (PD-1) and programmed cell death ligand-1 (PD-L1), and agents to inhibit these molecules have been approved by the FDA. PD-1 primarily regulates the proliferation of cytotoxic T lymphocytes, whereas CTLA-4 inhibits memory T cell activity^131^. PD-1 is expressed on T cells and has two ligands, PD-L1 and PD-L2. The expression of PD-L1 on tumor cells inhibits antitumor activity by binding to PD-1 on effector T cells. The combination of PD1 and CTLA4 antibodies is more effective than either antibody alone in treating a variety of cancers^134^. Anti-CTLA4 treatment enhances antigen-specific T cell-dependent immunity, while anti-PD-1 reactivates CD8+ T cell ability to kill cancer cells^134^.

Indoleamine 2,3-dioxygenase (IDO) is another possible immunotherapy target, as its inhibition potentiates chemotherapy^135^. IDO is an enzyme that catalyzes the rate-limiting step of the tryptophan to kynurenine pathway, and it also promotes Treg differentiation and dampens CD8+ T cell activation, thus contributing to an immunosuppressive environment. High levels of IDO expression are associated with poor outcomes in many cancers, including glioblastoma, and may contribute to resistance to immunotherapy^136^.

The clinical activity of checkpoint blockade correlates with three main variables^131^: (1) the number of nonsynonymous/frameshift somatic mutations in the tumor, which results in the production of “neoantigens”; (2) high expression of the PD-1 ligand in tumor cells; and (3) the frequency of activated CD8+ T cells in the circulation^137^.

Issues related to the use of immune checkpoint inhibitors remain and include immune-related adverse events (e.g., development of autoimmunity), treatment resistance, and clinical benefits limited to only a fraction of patients^129^. Chemotherapy and radiation may perturb this barrier and allow monoclonal antibodies to access the brain^138^. Biomarkers of the treatment response, such as the determination of PD-L1 expression by immunohistochemistry, CD8+ T cell infiltration and distribution at tumor margins, and a high mutational load, which correlates with the clinical response to anti-PD-1/PD-L1 treatment, need to be standardized to improve their utility.

There are currently five clinical trials on pediatric brain cancers in which immune checkpoint inhibition in combination with other therapeutics is being used (Table 2). The treatments represent a range of therapies. One trial is investigating indoximod, an IDO pathway inhibitor, along with temozolomide for pediatric patients with progressive primary malignant brain tumors in a first-in-children phase 1 trial (NCT02502708). Another trial is using pembrolizumab, a humanized monoclonal IgG4 antibody directed against the PD-1 receptor (NCT02359565), to prevent the binding and activation of PD-L1 and PD-L2. The third trial is using pidilizumab, an anti-PD-1 antibody, in combination with subsequent treatment with radiation and then cyclophosphamide chemotherapy in DIPG (NCT01952769). Other trials are using nivolumab, a human programmed death receptor-1 (PD-1)-blocking antibody combined with ipilimumab, which inhibits CTLA-4. Overall, most clinical trials on pediatric brain cancer are in phase I, and a few are in phase II.

Despite an initial report of success using checkpoint inhibitors with nivolumab in two pediatric patients^139^, several limitations to the trial were noted. These two patients were siblings with hypermutant glioblastoma associated with germline biallelic mismatch repair deficiency (bMMRD). Their success was not surprising since the high mutational burden, high prevalence of tumor neoantigens, and elevated DNA mutations in repair pathways are associated with a favorable response to immune checkpoint inhibitors^140^. Except for children with a hereditary mismatch repair mutation, pediatric brain tumors generally do not exhibit high mutational rates^97^. These cases may provide insight into the mechanisms responsible for the development of pediatric brain cancers; for example, up to 40% of pHGGs in Jordan were associated with MMRD and, therefore, are likely to have a high mutational burden, suggesting that they may respond to immunotherapy treatments^141^. Clinical trials on immune checkpoint inhibitors for pediatric brain cancers will be most impactful if they include patients both with and without high mutational burdens, as this will allow clinicians to determine the patient populations most likely to benefit from the treatment. Since, in general, clinical trials on immune checkpoint inhibitors are performed on patients treated with combination therapies, it is difficult to assess the disease response to novel therapies. The utility and success of checkpoint inhibition therapy for pediatric brain cancer remain to be demonstrated.

Unlike checkpoint inhibition, CAR-T cell therapy targets tumor-specific antigens (TSAs) and creates an active immune reaction against the tumor. It may therefore be useful against pediatric brain tumors, which generally do not carry a high tumor mutational load.

MHC1 downregulation in tumors is a significant mechanism of tumor immune escape and presents a major hurdle for immunotherapy^142^. Since CARs usually recognize unprocessed antigens presented on cancer cells, CAR T cells that recognize surface antigens can circumvent MHC downregulation by tumor cells^143–145^.

A groundbreaking 2016 study presented the first evidence that CAR-T cell therapy could induce brain tumor regression^142^. CARs are hybrid receptors that contain a fusion of a specific antibody-binding domain and the T cell signaling machinery. These engineered receptors were integrated into T cells from patients using retroviruses or lentiviruses and reinfused back into the patient to target TSAs^21^. Pediatric brain tumor patients may benefit from CAR-T cell therapy, as these tumors lack adequate levels of immune cells to mount a robust immune response.

Antigen escape and antigen downregulation are major issues that hinder the success of CAR T cell therapy^111^. Another hurdle is the immunosuppression of engineered T cells by a tumor. For these reasons, current development efforts combine CAR-T cell treatment with vaccines to reinvigorate T cell responses.

The five most recent clinical trials using CAR-T cell therapy for pediatric brain tumors (Table 2) are all in phase I. These therapies target different antigens, including HER2, IL13Rα2, EGFR, and B7H3. CAR-T cells are commonly given through an in-dwelling catheter directly into ventricles or the tumor cavity to bypass the BBB.

In a recent study, the delivery of targeted CAR-T cells directly into the cerebrospinal fluid of recurrent pediatric brain tumors was more effective than delivery via the bloodstream^146^. This approach bypasses the BBB and minimizes the exposure of the remainder of the body to CAR-T cells, which minimizes potential side effects. Some researchers found that the combination of CAR-T cells with the DNA methylation inhibitor azacytidine was more effective than either treatment alone. Patients are currently being recruited for a first-in-child clinical trial to test the safety and antitumor efficacy of direct CSF delivery (ClinicalTrials.gov identifier: NCT02442297)^147^.

Vaccine therapy aims to activate the immune system to facilitate tumor cell killing^88,148^. Antitumor vaccines are designed to overcome an immune system that has been trained to tolerate the tumor. There are multiple approaches to vaccine design; the specific methods are described elsewhere^149–154^.

Active vaccine immunotherapies utilize antigens to induce antibodies and immune responses through direct immune system stimulation. An active immunotherapeutic agent produces a durable response by inducing immunological memory similar to normal immune responses. Active immunotherapies can be nonspecific (e.g., the use of cytokines to stimulate an immune response^155^) or specific (e.g., directed at a specific tumor antigen). Passive immunotherapies administer antibodies directly to the system^152^ and produce an immediate effect but without engaging immune memory. Vaccine development is currently focused on peptide-, DC-, and nucleic acid (gene)-based technologies.

Peptide-based vaccines against tumor antigens are the most straightforward and are a common approach^153,156^. The peptide targets are based on either tumor-specific antigens (TSAs) or tumor-associated antigens (TAAs; antigens that are highly expressed in but not exclusive to tumors). TAAs and TSAs are heterogeneous; they can be common among patients with the same cancer type or unique to a particular patient^157^. Targeting a few specific TAAs can quickly lead to the development of epitope variants and even loss of expression of the target epitopes, rendering TAA vaccines ineffective (107). To overcome tumor escape and achieve clinical benefits, vaccines against multiple antigens will need to be used in combination with other agents, such as immunostimulatory adjuvants, therapies that release damage-associated molecular patterns, and immune checkpoint blockade, to overcome tumor escape and achieve clinical benefits^156^.

Although most pediatric tumors have a low mutational burden, they carry many alterations in epigenetic regulators that are potential targets, such as the re-expression of developmental antigens. For example, DIPGs often carry a mutation in histone 3.3 or 3.1 (K27M)^4,30,34^. H3.3K27M-specific cancer peptide vaccines are in early phase clinical trials for pediatric patients with H3.3K27M-positive DIPG (ClinicalTrials.gov: NCT02960230).

DCs are immune cells that present antigens to T cells to induce an antigen-specific response. DC vaccines are an active area of vaccine development^151,158^. Naive DCs are isolated from the patient, matured and stimulated with the antigen ex vivo, and fully mature, antigen-loaded DCs are injected back into the patient.

Gene-based vaccines do not depend on MHC expression in the patient since they use the patient’s cell machinery to express proteins for personalized processing. Antitumor DNA vaccines deliver genes encoding tumor antigens to improve the adaptive immune response^159^. DNA vaccines are currently limited by their poor performance in humans, likely because they require access to the nucleus^160^.

RNA vaccines are composed of an mRNA and template DNA that encodes the target antigen(s). RNA vaccines present many advantages, primarily that they can easily be produced in the laboratory and are less expensive to produce than conventional vaccines^161,162^. After being internalized by host cells, mRNA transcripts are translated in the cytoplasm, and then, similar to DNA vaccines, target antigens are presented on the cell surface to antigen-presenting cells, which stimulates a T cell response^162^. An essential challenge for RNA vaccines is the delivery method: free RNA in the body is rapidly broken down and impedes the effective delivery of RNA vaccines. Various methods of delivery are being explored, such as incorporation of the RNA strand into a larger molecule to stabilize it or by packaging the RNA vaccine into liposomes^162,163^.

Oncolytic virus therapy (virotherapy) is a newer immunotherapy approach^92^. The aim of oncolytic virus therapy is to deliver a live modified virus that specifically kills and lyses tumor cells^164–166^. Oncolytic viruses can be engineered to improve therapeutic efficacy, and their combination with other agents can synergize with their antitumor effects. Usually, oncolytic viruses are injected directly into the tumor or given by intravenous injection or within cellular carriers.

Oncolytic viruses have more advantages than standard therapies, including (1) the selective infection and replication in cancer cells, (2) the lack of resistance from tumor cells, (3) the virus spreads throughout the tumor after a few cells have been infected, and (4) the virus can trigger an immune response against the tumor^7–10^.

The use of viruses to treat cancer is not new, but molecularly engineered platforms are novel. Different viruses have different properties, and their specificity for tumor cell infection is accomplished by designing the virus such that it can replicate only in tumor cells. In a recent study, four viruses (Adenoviridae, Poxviridae, Herpesviridae, and Reoviridae) were evaluated in the context of adoptive T cell therapy^167^. In an in vivo immunocompetent tumor model of adoptive tumor-infiltrating lymphocyte therapy, adenovirus was the most effective therapy in synergy with T cell therapy^167^.

After infection with an oncolytic virus, cancer cells are killed by either direct oncolysis, apoptosis, or immune activation against the infected cell. Dying cancer cells often release novel neoantigens to the immune system, boosting the immune response against the remaining tumor cells^168^.

Although the results of cancer vaccine trials have been encouraging, obstacles remain. Cold tumors have low response rates to cancer vaccines. The advantage of virotherapy is that it destroys cancer cells directly or activates the immune system to kill cancer cells and does not rely entirely on a preexisting “hot” immune environment.

The antigen heterogeneity of tumors and immune escape present additional obstacles to creating effective cancer vaccines. Neoantigens in a tumor may be expressed on some tumor cells but not on others, which can cause some cells to escape immunotherapy^100^. To minimize tumor immune escape, patients should receive cancer vaccines that target multiple neoantigens^156^.

A growing number of clinical trials are evaluating vaccines to treat pediatric brain cancer. The examples in Table 2 show a range of vaccine types and combinations. All the trials are in phase I or phase II. The vaccines include peptides, tumor lysate-pulsed DCs, tumor RNA-pulsed DCs (CMV-DCs), oncolytics, and so forth. Current trials combine vaccines with a range of other treatments, such as radiation, adjuvants (e.g., poly-ICLC, KLM, and GM-CSF), checkpoint inhibitors (e.g., ipilimumab, nivolumab), monoclonal antibodies against EGFR (e.g., nimotuzumab) and other facilitators of the immune responses.

The BBB has historically limited drug delivery to the brain, but new advances in drug delivery strategies have bypassed the BBB to improve treatment efficacy. For example, intratumoral delivery via a convection-enhanced catheter is becoming more common for the delivery of viruses, pharmaceuticals, and cell-based therapies to bypass the BBB, particularly in DIPG patients^91,169,170^.

Sayour et al.^163^ developed an innovative lipid nanoparticle-delivered immunotherapy for pediatric brain tumors; the delivery method represents a considerable advance for gene-based vaccines. Unlike DC vaccines, which require weeks of processing, RNA nanoparticles (RNA-NPs) can be produced in a few days after tumor tissue is acquired. RNA-NPs are created by combining nanoliposomes with tumor-derived RNA, and they can be presented by MHC molecules to quickly activate the T cell response. Immunocompetent mouse models of HGG responded well to RNA-NP treatment^163^. Furthermore, when RNA-NPs were compared with total tumor RNA (TTRNA)-loaded DC vaccines in the mouse models, both were equally effective at stimulating CD8-positive T cells. The same investigators are conducting a recently initiated clinical trial (NCT03334305) on the TTRNA-DC vaccine; the vaccine is injected under the skin at several time points, and then xALT vaccine (tumor-specific T cells) is infused into the blood through a peripheral IV catheter.

Oncolytic viruses have significant potential for pediatric brain cancer treatment since they do not depend on a high mutation load^91^. G207 is an example of a neuroattenuated, replication-competent, engineered HSV-1 oncovirus (Table 2). G207 delivered to the cerebellum of a mouse model successfully targeted and treated an aggressive MYC-overexpressing group 3 murine medulloblastoma^171^. Oncolytic HSV-1 G207 targets glioma cells and can induce a tumor-specific immune response in addition to its cytotoxic effects on its target cells^172^. A successful phase I trial on malignant glioma demonstrated that single-dose oncolytic HSV therapy is safe and may be effective when combined with radiation^172^. A new phase II clinical trial on pediatric glioma (NCT04482933) was initiated using intracerebral administration.

## Summary

Recent advances in genomics and molecular profiling have demonstrated that pediatric and adult CNS tumors differ from one another, yet the treatments for CNS tumors were designed for and tested in adults, reducing their efficacy for pediatric CNS tumors. Unlike adult CNS tumors, pediatric CNS tumors generally have a low mutational load and a low level of neoantigens, and pediatric CNS tumors also have epigenetic profiles that differ from those in adult CNS tumors. These factors present obstacles to providing effective care for pediatric CNS tumor patients, as the available epigenetic modifiers and immunotherapies are often ill-suited for pediatric CNS tumors. However, recent efforts have shown promise—there are many ongoing clinical trials on pediatric CNS cancers that combine patients across multiple cancer centers and hospitals, which will improve the statistical power of the clinical trials. Additionally, combining immunotherapy with standard treatments has improved efficacy against pediatric CNS tumors. Our understanding of the signaling mechanisms in the tumor microenvironment has also improved our treatment options for pediatric CNS tumors, as clinicians can better tailor treatment regimens to individual patients for better outcomes.

## Future directions

We have made major advances in some subgroups of pediatric CNS tumors, such as medulloblastomas, but the prognoses of other pediatric brain tumors, such as HGGs and DIPGs, remain largely unchanged. Epigenetics and immunotherapy are providing many new and exciting opportunities to improve brain cancer treatment that need to be extensively validated in real-world settings. Novel options that bypass the need for neoantigens, such as oncolytic virus therapy, are promising, particularly when included in combination approaches. While modulating immune inhibitory pathways has been considered a significant breakthrough in cancer treatment in clinical trials, more data are needed to evaluate the success rates of these novel therapies, as they often require a personalized approach towards treatment depending on the molecular profile of the tumors. While immune checkpoint blockade is a promising approach, the addition of epigenetic modulators, such as HDACis or DNMTis, to immunotherapy with CAR-T cells or oncolytic viruses also needs to be substantiated for efficacy and safety in a real-world setting.

Current research aims to identify biomarkers that will reliably predict a patient’s response to checkpoint blockade inhibitors and whether the patient is likely to become resistant to treatment. It is expected that genetic profiling (mutations and epigenetic modifications) will provide a robust and accurate molecular characterization system. Integration of these new profiling systems as a clinically feasible tool in the diagnosis and management of pediatric brain tumors is expected to not only complement standard therapies currently in use for pediatric brain tumor patients but also help improve clinical trial designs, especially while profiling patients during recruitment for clinical trials.

Currently, there is a broad array of treatment approaches in clinical trials that will hopefully allow standardized procedures based on individual tumor profiles. Future progress towards the development of combination approaches is expected to involve progress in biomarker detection to personalize treatment and monitor treatment progress, as well as to develop algorithms for therapeutic combinations. One of the key areas where significant progress in clinical research is required includes the process of validation and systematic analyses of biomarkers and immune correlates in both adults and children.
